# Acute liver failure complication of brucellosis infection: a case report and review of the literature

**DOI:** 10.1186/s13256-018-1576-4

**Published:** 2018-03-09

**Authors:** Julio César García Casallas, Walter Villalobos Monsalve, Sara Consuelo Arias Villate, Ingrid Marisol Fino Solano

**Affiliations:** 1 Internal Medicine and Clinical Pharmacology, Department of Clinical Pharmacology and Therapeutics, Clínica Universidad de La Sabana, Chía, Colombia; 2 Pharmacology Department, University of La Sabana, Chía, Colombia; 3 Universidad El Bosque, Chía, Colombia; 4 Research Group Therapeutic Evidence, University of La Sabana, Chía, Colombia; 5 Internal Medicine, Clínica Universidad de La Sabana, Chía, Colombia; 6 Clinical Pharmacology Service, University of La Sabana, Chía, Colombia; 7 Clinical Pharmacology Resident, Clinical Pharmacology Department, University of La Sabana, Chía, Colombia

**Keywords:** Brucellosis, Acute liver failure, Zoonosis, Parasitic infections

## Abstract

**Background:**

Brucellosis is one of the most widespread zoonoses worldwide. It can affect any organ system, particularly the gastrointestinal system, but there is no report of acute liver failure as a brucellosis complication.

**Case presentation:**

We present a case of acute liver failure secondary to brucellosis infection. A 75-year-old Hispanic man presented to a University Hospital in Chía, Colombia, with a complaint of 15 days of fatigue, weakness, decreased appetite, epigastric abdominal pain, jaundice, and 10 kg weight loss. On examination in an emergency room, abdomen palpation was normal with hepatosplenomegaly and the results of a liver function test were elevated. The diagnosis of brucellosis was confirmed by epidemiological contact and positive Rose Bengal agglutination with negative enzyme-linked immunosorbent assay immunoglobulin M for *Brucella*. He was then treated with doxycycline plus trimethoprim/sulfamethoxazole, with a favorable clinical outcome.

**Conclusions:**

The clinical presentation of brucellosis can be very imprecise because it can affect any organ system; however, there is no report of acute liver failure as a brucellosis complication. This is the first reported case in the Colombian literature of acute liver failure due to brucellosis. We found this case to be of interest because it could be taken into account for diagnosis in future appearances and we described adequate treatment and actions to be taken at presentation.

## Background

*Brucella* species are bacteria that infect humans as an incidental host. Human brucellosis usually results from contact with fluids from infected animals (cattle, sheep, goats, pigs, dogs, and rats) or by consumption of contaminated animal products such as unpasteurized milk and cheese [1]. This infection has multiple clinical manifestations and requires a specific diagnosis based on epidemiological analysis and laboratory confirmatory tests in order to initiate the appropriated antibiotic treatment. Here we present the case of a patient with brucellosis infection secondary to contact with cows from his farm infected with brucellosis. He presented with acute liver failure (ALF) which is a rare but possible complication of this zoonosis.

## Case presentation

A 75-year-old Hispanic man, born and resident in Zipaquirá, Cundinamarca (which is a Colombian rural area), who works as an independent seller, presented to the emergency department of the Clinic of the Universidad de La Sabana with a complaint of 15 days of fatigue, weakness, decreased appetite, epigastric abdominal pain, jaundice, and 10 kg weight loss. He had a history of peptic disease and degenerative arthritis.

On examination in our emergency department his temperature was 36.5, his blood pressure was 110/70 mmHg, heart rate 103 beats per minute, respiratory rate 22 breaths per minute, and oxygen saturation was 92%. His skin was pale and he had mucocutaneous jaundice, icteric conjunctivae, and bleeding stigmata of mucosa in oral cavity. Abdomen palpation revealed tenderness at palpation in right and left upper quadrants and epigastric region. His liver edge was smooth and was palpated 2 cm below the right costal margin; his spleen edge was palpated 2 cm below left costal margin. His reflexes were 1 out of 4 and there was no clonus. The remainder of the physical examination was normal.

His hemoglobin, hematocrit, and platelet counts were normal. His white blood cells and liver function test were elevated and coagulation times were prolonged; other laboratory results are shown in Table 1.

**Table 1:** Laboratory results of the patient

Laboratories	Result						Reference value
	At presentation	Day 2	Day 3	Day 5	Day 7	Day 8	
Hemoglobin	15.2	13.7	11.6	13.1	11.4	11.2	12–16 gr/dl
Hematocrit	43	40	33	38	34	33	36–43%
Platelets	38.700	21.000	37.200	48.200	63.400	80.000	140–440 × 10^3^ × mm^3^
Leukocytes	32.160	12.160	7.600	6.560	8.370	10.330	4.5–10.5 × 10^3^ × mm^3^
Neutrophils	19	42		31	51	32	35–70%
Lymphocytes	35	49		75	35	10	20–45%
INR	2.13		1.82	1.41		1.15	
PTT		52.1	44.9	39.5	36.8	30.5	Control 30 seconds
Total bilirubin	35	15.9	13.2	10.4	4.4	4.5	0–1 mg/dl
Direct bilirubin	27.5	12.4	11.0	8.5	3.4	3.2	0–0.3 mg/dl
Indirect bilirubin	7.7	2.8	2.2	1.9	0.87	1.30	0–0.7 mg/dl
AST (SGOT)	53	48	65	62	53	45	0–32 UI/ml
ALT (SGPT)	57	56	50	44	62	56	0–31 UI/ml
Alkaline Phosphatase	616		665			613	35–104 UI/ml
Creatinine	1.05		2.3	1.62	1.6	1.3	0.4–1.4 mg/dl
Urea nitrogen	13.9		61.9	42.3	41.0	38.5	4.6–23.4 g/dl
Albumin		1.9					3.4–4.8 gr/dl
Potassium		4.6		3.97	4.4		3.5–4.5 mEq/L
Sodium		136	138	135	138		135–148 mEq/L
Calcium		7.4					8.6–10.2 g/dl
Chlorine		105	108				98–107 mEq/L
DHL		459					240–480 UI/ml
GGT		226					5–39 UI/L
Glycemia			74				70–110 mg/dl
HIV serology				Negative			
Blood cultures				Negative			
Peripheral blood Smear	Microcytosis, mild anisocytosis. White line with lots of reactive lymphocytes. Hypochromia						

*ALT* alanine aminotransferase, *AST* aspartate aminotransferase, *DHL*, *GGT* gamma-glutamyltransferase, *INR* international normalized ratio, *PTT* partial thromboplastin time, *SGOT* serum glutamic oxaloacetic transaminase, *SGPT* serum glutamic pyruvic transaminase

**Fig. 1:**
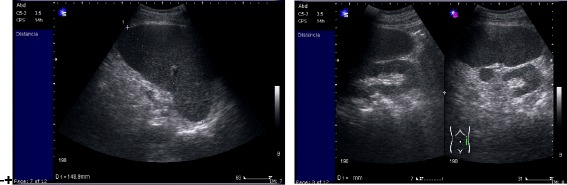
Upper abdominal ultrasound

**Fig. 2:**
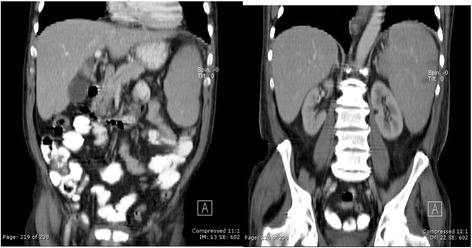
Computed tomography of the abdomen with contrast

An upper abdominal ultrasound showed normal-sized liver, normal liver shape, no focal or diffuse lesions observed in the parenchyma, distended gallbladder with wall thickening of 4 mm, no evidence of biliary stones inside, and significant splenomegaly of approximate 17.5 cm, without focal lesions (Fig. 1). Computed tomography (CT) of his abdomen, with the administration of contrast material, showed diffuse heterogeneous attenuation in his liver, and splenomegaly with contrast (Fig. 2).

He was seen by surgery and gastroenterology services who considered support for viral hepatitis or acute cholangitis with ALF and decided to start antibiotic treatment with ampicillin/sulbactam 3 gr every 6 hours. The next day there was an increase in our patient’s liver function test results and his kidney test results started to elevate. He was re-interrogated by medical attendants; he told them that he had been in contact during the last 2 months with a cow who died of brucellosis; the diagnosis of the cow had been made by a specialist vet. With this background, the physician suspended the antibiotic scheme and added doxycycline 200 mg every day plus trimethoprim/sulfamethoxazole (TMP/SMX) 80/400 mg every 6 hours.

A sero-agglutination test Rose Bengal was reported as positive and enzyme-linked immunosorbent assay (ELISA) immunoglobulin (Ig) M for *Brucella* was negative.

The evolution after the onset of specific antibiotic treatment was favorable with evident clinical improvement of his symptoms and paraclinical tests. He could go home after 11 days of hospitalization. The discharge diagnosis was ALF secondary to brucellosis. A month later our patient was followed up with a hepatic scintigraphy, where only mild cirrhosis was reported; in addition, he was asymptomatic and without clinical signs of sequelae or reinfection.

## Discussion

*Brucella* species are a Gram-negative coccobacillus, aerobic, non-spore forming, and non-motile bacteria. There are six recognized species according to their primary host preferences, that is *Brucella abortus*, cattle; *Brucella melitensis*, in sheep and goats; *Brucella suis*, in pigs; *Brucella ovis*, in sheep; *Brucella canis*, dogs, and *Brucella neotomae*, in wood desert rats; however, there is no clear basis for host preferences although there may be pseudogenes that influence host adaptation. *Brucella* infects humans as an incidental host after direct contact with tissues or blood from infected animals or by consumption of contaminated animal products like unpasteurized milk and cheese [1–3].

The prevalence of *Brucella* infection in humans depends on factors such as husbandry practices, food preparation techniques, and trade of animals. There are no preferences for age and sex. The current seroprevalence rate for brucellosis in cattle in Colombia is estimated between 2.4 and 5%, especially in rural areas [4]. Infection may also result from the entry of the bacteria from infected animals through skin lesions, conjunctiva, or from inhalation [5, 6]. In the pathogenesis of *Brucella*, *Brucella* are ingested by polymorphonuclear cells and macrophages, then move to local lymph nodes to replicate intracellularly, and bacteria from lysed cells can infect other cells or disseminate systemically. During the intracellular phase, *Brucella* display survival strategies to suppress host’s immune response and avoid destruction; all these activities may promote the chronicity of infection [1, 7].

The incubation period is approximately 1 to 4 weeks. Brucellosis is a systemic infection with a broad clinical spectrum from asymptomatic disease to severe or fatal illness. The main presentations are insidious onset of fever, arthralgia, myalgia, weight loss, and abdominal pain as well weakness, fatigue, and headache. Physical findings are variable, such as hepatosplenomegaly or lymphadenopathy; although these are very unspecific findings, we correlated clinical manifestations with the epidemiologic history referred by our patient in this case. Brucellosis can develop into a chronic disease and be persistent, becoming a granulomatous disease capable of affecting any organ system [2, 8].

In approximately 30% of cases, brucellosis can affect any organ system [9]:

- Genitourinary system – the most common manifestation is orchitis and epididymitis [9].
- Hematological system – it can cause anemia, leukopenia, thrombocytopenia, and disseminated intravascular coagulation [6].
- Neurological system – it includes meningitis, encephalitis, and neuritis [10].
- Osteoarticular system – is the most common presentation, it could involve sacroiliac joints and large joint of lower limbs [11].
- Pulmonary system – it could cause bronchitis, interstitial pneumonitis, lobar pneumonia, pleural effusion, or empyema [12].

The gastrointestinal system can be compromised; a patient can present with clinical hepatitis and other manifestations such as hepatic or splenic abscess, pancreatitis, colitis, and spontaneous peritonitis, which are rare [13]. Liver involvement is frequent in acute and chronic brucellosis, as an increase in transaminase values and a mild hepatosplenomegaly can occur; sometimes an acute hepatitis develops [14]. However, ALF is a rare condition that happens in previously healthy individuals without pre-existing cirrhosis in which there is a rapid deterioration of liver function results, coagulation abnormality, usually an international normalized ratio (INR) > 1.5, and any degree of mental alteration (encephalopathy); all of this was presented clinically by our patient [15].

In this case report, the epidemiology and patient demographics, clinical presentation, laboratory findings, and diagnostic images, ruled out the possible presence of toxins, viral hepatitis, vascular events, and miscellaneous conditions as a possible cause of ALF, leading to the increasing diagnostic probability of the findings being attributed to brucellosis [4]. Regarding the diagnosis, there are methods to detect antibodies against cell wall components or cytoplasmic proteins of the bacteria, for example: serum agglutination, ELISA, Rose Bengal agglutination, Coombs test, Immunocapture agglutination (Brucellacapt®), and 2-mercaptoethanol agglutination [16].

ELISA is the second most common serologic method after the serum agglutination test. ELISA is objective, rapid, and highly sensitive; it measures IgM, IgG, and IgA. However, there are problems with the examination, for example, quality and interpretation of results could be different in different laboratories [16, 17]. There are investigations that concluded that ELISA does not improve diagnostic accuracy compared with other techniques, and they showed that the sensitivity to IgM or IgG was lower compared to serum agglutination. Other reports described negative ELISA with multiorgan compromise [18]. This could be an explanation of the negative ELISA IgM report in this case report.

Rose Bengal agglutination is a cheap, effective, and rapid slide-type agglutination serologic test performed with a stained *B. abortus* suspension at pH 3.6–3.7 and plain serum. Because of its simplicity, it is often used as a screening test in human brucellosis and would be optimal for small laboratories with limited means [19, 20].

The standard treatment for brucellosis is to control the illness and prevent complications and relapses. The goal of disease management is based on appropriate antibiotic therapy that includes drugs that can penetrate macrophages and act in the intracellular acidic medium [21]. There are two major regimens for the treatment:

1. Doxycycline 100 mg orally twice a day for 6 weeks, plus gentamicin 5 mg/kg daily for 5 to 14 days [21, 22].
2. Doxycycline 100 mg orally twice daily plus rifampin 600 to 900 mg (15 mg/kg) orally once daily for 6 weeks [21].

For the case presented in this case report, management was associated with doxycycline + TMP/SMX, which is considered in the literature to be an optional scheme for the treatment [23–25]. Our patient had an adequate response to this management.

## Conclusions

To the best of our knowledge, this is the first reported case in the Colombian literature of ALF due to brucellosis. In addition, ALF is not a common complication of a brucellosis infection. We found this case to be of interest because it could be taken into account for diagnosis in future appearances and we described adequate treatment and actions to be taken at presentation to control the illness and prevent complications and relapses.

## Abbreviations

ALF: Acute liver failure
CT: Computed tomography
ELISA: Enzyme-linked immunosorbent assay
Ig: Immunoglobulin
INR: International normalized ratio
TMP/SMX: Trimethoprim/sulfamethoxazole
